# UV-C and hydration state drive pulsed light-induced proteome damage in *Bacillus pumilus* spores

**DOI:** 10.3389/fmicb.2025.1579161

**Published:** 2025-04-09

**Authors:** Imed Dorbani, Jean Armengaud, Frédéric Carlin, Catherine Duport

**Affiliations:** ^1^Avignon Université, INRAE, UMR SQPOV, Avignon, France; ^2^Claranor SA, Avignon, France; ^3^Département Médicaments et Technologies pour la Santé (DMTS), Université Paris Saclay, CEA, INRAE, Bagnols-sur-Cèze, France

**Keywords:** pulse-light, UV-C, proteome, spores, *Bacillus pumilus*

## Abstract

**Introduction:**

Pulsed light (PL) is a non-thermal processing technology that inactivates microorganisms through high-intensity pulses of polychromatic light, including ultraviolet-C (UV-C). While the germicidal effect of PL has been widely studied, its impact on microbial proteomes remains poorly understood. Here, we investigate the proteomic response of *Bacillus pumilus* DSM492 (ATCC 27142) spores to PL treatment, comparing it to conventional UV-C 254 nm exposure.

**Methods:**

*B. pumilus* spores were either suspended in water or sprayed onto a polystyrene surface and exposed to PL or UV-C at fluences achieving a 5-log and a > 7-log reduction in viability. Proteomic changes were analyzed using mass spectrometry to identify proteins with decreased abundance after treatment.

**Results:**

PL treatment induced a significantly greater proteomic alteration compared to UV-C, particularly in spores suspended in water, where the number of proteins with decreased abundance was ~6-fold higher than in spores sprayed on a polystyrene surface. Proteomic analysis revealed that the effect of PL in water was primarily due to UV-C 254 nm, whereas on polystyrene, UV-C 254 nm had no significant impact. Furthermore, proteins most affected by PL were enriched in photosensitive amino acids such as tryptophan, histidine, tyrosine, cysteine, and methionine, suggesting oxidation and photoreactivity as key degradation mechanisms.

**Discussion:**

Although the overall inactivation rate could not be directly correlated with proteome damage, we identified that core proteins involved in DNA and RNA protection and repair were specifically targeted by PL. These findings provide new insights into the molecular mechanisms underlying PL-mediated microbial inactivation and highlight the role of protein photodamage in spore susceptibility.

## Introduction

Bacterial spores are remarkably resilient dormant cells, developed in response to adverse conditions (Setlow, 2014b). Their resilience, attributed in particular to a unique multilayer structure and the presence of specific spore molecules, allows them to endure a diversity of inactivation processes (Setlow and Christie, 2023). Following survival, these spores can rapidly germinate into vegetative cells when exposed to favorable environments (Setlow, 2014a). The proliferation of vegetative cells can have health, food safety and food quality implications. Therefore, ensuring the effective inactivation of spores and the prevention of germination and outgrowth is of major importance in health care facilities and in the food industry (Farag et al., 2021).

Many inactivation processes that are effective against vegetative cells are far less effective against bacterial spores, with large discrepancies in inactivation efficiency (Wood and Adrion, 2019). One effective method for spore inactivation is pulsed light (PL) technology (Levy et al., 2012). PL works on the principle of inactivating microorganisms using one or more flashes of white light. The short duration (around 250 μs) PL flash is characterized by high radiant flux (>10^3^ W/cm^2^) and a broad polychromatic spectrum (200–1,100 nm), encompassing UV, visible, and near-infrared spectra. The UV spectrum includes UV-C (200–280 nm), UV-B (280–315 nm) and UV-A (315–340 nm). The efficiency of PL irradiation depends on the applied fluence (energy in joules divided by the unit area treated, J/cm^2^), depending itself on the lamp charging voltage, the distance between the sample and flash-lamp, as well as the number of applied light flashes (Kramer et al., 2017a; Mandal et al., 2020). However, the mechanisms behind the killing effect of the fluence delivered by PL are still not fully understood.

PL inactivation of microorganisms involves primarily a photochemical effect, causing DNA, RNA and protein damages (Gómez-López et al., 2007; Kramer et al., 2017b; Santamera et al., 2020). A photothermal effect leading to structural changes has also been detected. These mechanisms can act independently or in combination. Thus, in addition to the tremendous energy delivered by PL flashes, PL may trigger other mechanisms beyond UV-C in microbial inactivation. The importance of photothermal and photochemical mechanisms may vary depending on fluence and the target organism. Additionally, a third effect, the photophysical effect, involving structural cell damage, was also described (Gómez-López et al., 2007; Santamera et al., 2020). The photochemical effect induces DNA damage similar to UV such as double- and single-strand DNA, and RNA breaks, and dimerization of pyrimidine bases in DNA (Heinrich et al., 2016). Photochemical-induced damage to proteins can also occur through direct absorption by specific amino acid residues (e.g., tryptophan, tyrosine, phenylalanine, histidine, methionine, and cysteine) (Pattison et al., 2011). PL also generates reactive oxygen species (ROS) that can target various molecules such as DNA, proteins, and lipids (Kramer et al., 2015). ROS are preferentially generated by UV-A and thus indirectly contribute to microorganism inactivation (Heinrich et al., 2016). Factors contributing to PL resistance include: (i) repair mechanisms, (ii) structural factors such as membrane and cell wall composition, or the presence of specialized pigments capable of absorbing certain wavelengths of light lethal to microorganisms (Setlow and Christie, 2023). Factors underlying differences between spore and vegetative cells include dipicolinic acid, α/β-type small acid-soluble proteins, spore coat proteins such as CotA, GerE, CotE, and CotG (Clair et al., 2020), and efficient DNA repair mechanisms (Esbelin et al., 2016).

The assessment of inactivation process efficacy is often conducted using a classic microbiological method known as the challenge test. This involves exposing the target microorganism to inactivation treatments and determining its survival capacity based on the applied parameters. In the case of PL inactivation, assessing microorganism resistance can be done through various methods. Typically, in research studies, microorganisms are suspended in water or buffered solutions like tryptone salt or phosphate-buffered saline and exposed to treatments (Bolton and Linden, 2003; Takeshita et al., 2003; Esbelin et al., 2016; Clair et al., 2020). Microorganism exposure can also occur on inert surfaces such as glass, stainless steel, or polystyrene (PS) (Levy et al., 2012; Wood and Adrion, 2019; Racchi et al., 2021). Two methods for inoculating a spore suspension onto a PS surface have been described: (i) spray inoculation; and, (ii) droplet deposition (Levy et al., 2011). Both methods share the advantage of directly assessing microorganism resistance without interference from water or other environmental elements.

Our previous research focused on the structural alterations and degradation of coat proteins under PL exposure (Clair et al., 2020), given their critical role in spore resistance. However, a broader investigation encompassing the entire spore proteome is warranted, especially considering the extensive degradative effects associated with UV technologies. Notably, research addressing industry-relevant fluences—those required to achieve approximately a 5-log spore reduction—remains scarce. Furthermore, while PL’s efficacy is often attributed to UV-C radiation, few studies have substantiated this claim at the proteomic level (Levy et al., 2012). Additionally, the potential variability in proteomic responses depending on PL methodologies is frequently overlooked in existing literature.

The present study rigorously examined the spore proteome profiling of *B. pumilus* DSM492, a crucial strain used for bio-validation in industrial sterilization systems (Prince, 1976), in response to PL. Specifically, we investigated the effects of PL at a fluence sufficient to achieve a 5-log reduction in spore counts, as well as at twice this fluence, which led to a >7-log reduction. Our primary objective was to determine whether PL-induced proteomic changes parallel those induced by UV-C at 254 nm. Furthermore, we compared the proteomic responses of spores suspended in water versus those sprayed on polystyrene surfaces (PS). These comparisons are pivotal for elucidating the mechanisms underlying spore inactivation by PL at the protein level.

## Materials and methods

### *Bacillus pumilus* spore preparation

The strain used in this study is *B. pumilus* DSM492 (ATCC 27142). To prepare purified bacterial spores, precultures were inoculated with a small amount of cell stock culture into L-Broth (LB) medium, incubated at 30°C for 16 h, and then spread onto fortified nutrient agar (FNA) sporulation medium (Fernández et al., 1999). After 7 days of incubation at 30°C, the spores were harvested from the agar surface and washed with deionized water six times through successive centrifugation steps (2 × 15 min at 7,000 g, 5,000 g, and 4,000 g, respectively), with the supernatants discarded and the spores resuspended in 2-mL demineralized water. The spore suspensions were then subjected to a heat treatment (80°C, 10 min) to inactivate vegetative cells. A spore yield of over 95%, characterized by phase-bright spores observed under phase-contrast microscopy, was achieved for all samples. The spore concentration was determined by spreading 100 μL of decimal dilutions onto plate count agar (PCA). The spore suspensions containing approximately 10^10^ colony forming units per ml (CFU/mL) were stored in darkness at 4°C in sterile distilled water until use.

### Pulsed light, and UV-C (at a wavelength of 254 nm) devices

The PL device comprised a stainless-steel enclosure housing an optical system consisting of three xenon lamps along with their optical reflector system (Levy et al., 2012; Esbelin et al., 2016; Clair et al., 2020; Dorbani et al., 2024b). Additionally, it included an adjustable lab-elevator mechanism for positioning samples beneath the lamps at the desired distances that determine the target fluences. The optical system was composed of three polished aluminum reflectors, one for each lamp, with a designed profile to ensure nearly uniform irradiance across a 10 cm x 10 cm square area. Fluence homogeneity was verified, and the relative standard deviation was less than 7.2% across the width of the treated area. The PL equipment emits short-duration pulses (250 μs) of polychromatic light covering a broad spectrum (200–1,100 nm) generated by xenon flash lamps. Testing was conducted with a charging voltage of 2,500 V. Spore samples were exposed to PL fluences adjusted by varying both the number of flashes (at one-second intervals) and the distance between the xenon lamps and the samples using the lab-elevator (ranging from 20.3 cm to 2.4 cm). Fluence measurements were taken using a Gentec joulemeter equipped with an UP-17 sensor designed for high-power measurements (Gentec-eo Electro-Optics Inc., Quebec, Canada) and connected to a display device (MAESTRO, Gentec-eo).

For UV-C exposure, a UVC chamber was fitted with two low-pressure mercury lamps, each measuring 1.6 cm in diameter and 30 cm in length (G5-TUV 8 W germicidal UVC, PHILIPS), emitting germicidal UV at a peak of 253.7 nm as per the manufacturer’s specifications. These lamps were positioned in parallel at a distance of 16.5 cm from the spore samples. The irradiance delivered under experimental conditions was quantified using the Gentec-eo joulemeter, equipped with an UP-12E Gentec-eo detector and connected to the MAESTRO display device. Fluence is defined as the product of irradiance and exposure time. Fluence homogeneity was verified, with a relative standard deviation of less than 3.2% across the width of the treated area.

### Inactivation of spores suspended in water

One milliliter of a suspension containing approximately 5 × 10^7^ CFU/mL of *B. pumilus* spores was deposited onto a Petri dish with a diameter of 55 mm and then manually spread over the entire surface. The dish was gently shaken, resulting in a 0.42 mm-deep spore suspension (i.e., the height of a 1 mL-volume and in a 55 mm diameter cylinder) spread over the whole Petri dish surface. The spores were then exposed to PL or UV-C radiation at 254 nm, as described previously (Dorbani et al., 2024b). Viability log-reduction values for each treatment condition were determined by counting the survivors capable of germination and colony formation on PCA plates.

### Inactivation of spores sprayed on polystyrene surface Petri dishes

The spores were sprayed onto 90 mm diameter PS Petri dishes using a previously described airbrush method with minor modifications (Levy et al., 2011). Briefly, 100 μL of a spore suspension containing approximately 10^10^ CFU/mL were loaded into a 2 mL Mecafer AG-1 airbrush reservoir (Mecafer SA, Valence, France) and sprayed from a distance of about 20 cm onto vertically placed sterile Petri dishes perpendicular to the spray direction. Ensuring the deposition of sprayed cells onto the dish bottom rather than the dish wall was facilitated by a Petri dish holder specifically designed for this purpose. Following spraying, the inoculated Petri dishes were air-dried for 4 h at room temperature, then exposed to PL fluences of 1.24 and 2.48 J/cm^2^ or UV-C fluences of 0.08 and 0.17 J/cm^2^. Spores deposited on unexposed Petri dishes (control) and Petri dishes exposed to PL and UV-C were placed with 50 mL of Ringer’s solution and Tween 80 (0.01%) in sterile plastic bags (Stomacher, UK) and subsequently detached by manual agitation for 1 min. The suspension was then transferred into a sterile 50 mL Falcon tube. Spore titers were determined through serial dilutions spread on PCA plates.

### Protein extraction from spores

After exposure to PL and UV-C, the spores were pelleted by centrifugation at 16,000 g for 5 min. They were then resuspended in 400 μL of denaturing buffer from the Minute^™^ Extraction Kit, YT-015 (Invent Biotechnologies, INC), and heated to 100°C for 5 min before being mechanically lysed using a Fast-Prep24™ high-speed benchtop homogenizer. Eight cycles of homogenization (40 s per cycle) were performed. Following homogenization, the lysate was centrifuged at 5,000 g for 15 min to separate the cellular debris, and the supernatant containing the proteins was collected. Subsequently, the supernatant underwent another centrifugation step at a higher speed (16,000 g) for 1 min to ensure complete recovery of the proteins it contained. Protein quantification was performed using a BiCinchoninic acid Assay (BCA) assay.

### Shotgun proteomic analysis

Proteins derived from untreated and PL- and UV-C -treated *B. pumilus* spores underwent short electrophoretic migration on NuPAGE 4–12% Bis-Tris gels (Invitrogen), using NuPAGE MES supplemented with the antioxidant NuPAGE as the running buffer (Rubiano-Labrador et al., 2014). The proteins were digested in-gel with trypsin (Roche) following the ProteaseMAX protocol (Promega). The samples underwent reverse phase liquid chromatography–tandem mass spectrometry (nanoLC-MS/MS) on a Q-Exactive HF mass spectrometer coupled with an Ultimate 3000 nano LC system (Thermo Electron, Villebon-sur-Yvette, France). The nanoLC-MS/MS analysis proceeded as follows: peptides were loaded for online desalting onto an Acclaim PepMap 100 C18 reverse-phase pre-column (100 Å pore size, 300 μm inner diameter × 5 mm), then resolved over 90 min on an Acclaim PepMap 100 C18 nanoscale column (3 μm bead size, 100 Å pore size, 75 μm i.d × 500 mm) at a flow rate of 200 nL/min. Trypsin-digested MS/MS spectra were searched against the *B. pumilus* ATCC 27142 protein sequences downloaded from the NCBI database, using the MASCOT Daemon search engine with the following parameters: 5 ppm peptide mass tolerance, 0.02 Da MS/MS fragment mass tolerance, peptide charge 2^+^ or 3^+^, a maximum of two missed cleavages, carbamidomethylation of cysteine (+57.0215) as a fixed modification, and methionine oxidation (+15.5949) as a variable modification. Only peptides identified with a *p*-value ≤ 0.05 in homology threshold mode, and proteins identified by at least two distinct peptides, were retained during parsing with the IRMa v1.3.1 software, following recommendations (Christie-Oleza et al., 2012). The false discovery rate determined from the corresponding decoy database was estimated to be less than 1%. The number of MS/MS spectra assigned to each protein (spectral count) was determined as previously described (Cogne et al., 2019). Protein abundance was compared between untreated spore samples (controls) and those treated with PL or UV-C using the TFold test (Carvalho et al., 2012). Proteins were considered significantly impacted by treatment compared to the control when their abundance decrease was greater than 1.5-fold ((|log_2_ fold-change| ≥ 0.59)) and the *p*-value ≤ 0.05. The mass spectrometry proteomics data have been deposited to the ProteomeXchange Consortium via the PRIDE partner repository with the dataset identifier PXD052144 (DOI: 10.6019/PXD052144).

### Functional annotation and enrichment analysis

To explore whether certain biological processes are enriched among the proteins that were found significantly changed with the treatment, we used the comprehensive bioinformatics tool for functional annotation Swiss-Prot/TrEMBL,^1^ and String,^2^ and the *Bacillus pumilus* SAFR-032 strain as a reference.

### Bioinformatics and statistical analyses

Venn diagrams of the proteins were created using Venny online software,^3^ to select the common proteins from among the analyses. Principal component analysis (PCA) was performed using ClusVis.^4^ GO analysis was performed using ShinyGo 0.80.^5^ Statistical analyses were performed using GraphPad Prism software version 5.0.

## Results

### Overview of proteomic analyses

We aimed to compare the effects of PL and UV-C on the proteome of *B. pumilus* spores by selecting irradiation conditions that resulted in the same level of log reduction for spores suspended in water or sprayed on PS Petri dishes. Specifically, we chose to compare the effects at fluences that led to a 5-log reduction and then tested the effects when these fluences were doubled. To achieve this, the spores of *B. pumilus* suspended in water (5 × 10^7^ CFU/mL) were exposed to fluences of 1.31 J/cm^2^ (noted *F*_5_ as the fluence allowing for a 5-log reduction) and 2.62 J/cm^2^ (noted 2*F*_5_, as twice the fluence allowing for a 5-log reduction) of PL, and 0.10 J/cm^2^ (*F*_5_) and 0.20 J/cm^2^ (2*F*_5_) of UV-C. The spores sprayed on PS Petri dishes (1 × 10^8^ CFU/mL) were exposed to fluences of 1.24 (*F*_5_) and 2.48 (2*F*_5_) J/cm^2^ of PL, and 0.08 (*F*_5_) and 0.16 (2*F*_5_) J/cm^2^ of UV-C. For spores suspended in water, the *F*_5_ fluences caused a 5.18 ± 0.34 log reduction (mean ± standard deviation; *n* = 3), and a 5.23 ± 0.49 log reduction (*n* = 3) for PL and UV-C, respectively. The log reduction by PL or by UV-C at 2 *F*_5_ was greater than 7.75 ± 0.08 (Supplementary Table S1). For spores sprayed on PS Petri dishes, the *F*_5_ fluence caused a 5.20 ± 0.33 log reduction (*n* = 4) by PL, and a 5.18 ± 0.15 log reduction (*n* = 4) by UV-C. The log reduction at 2 *F*_5_ was 7.45 ± 0.04, and 7.01 ± 0.22 by PL and UV-C, respectively (Supplementary Table S2) (Dorbani et al., 2024b). The morphology of *B. pumilus* spores was examined following exposure to PL and UV-C, using both phase contrast microscopy and scanning electron microscopy. No significant morphological changes were observed in treated spores compared to untreated spores (data not shown). These results indicate that neither treatment induced visible structural alterations at the cellular or ultrastructural levels.

**Figure 1:**
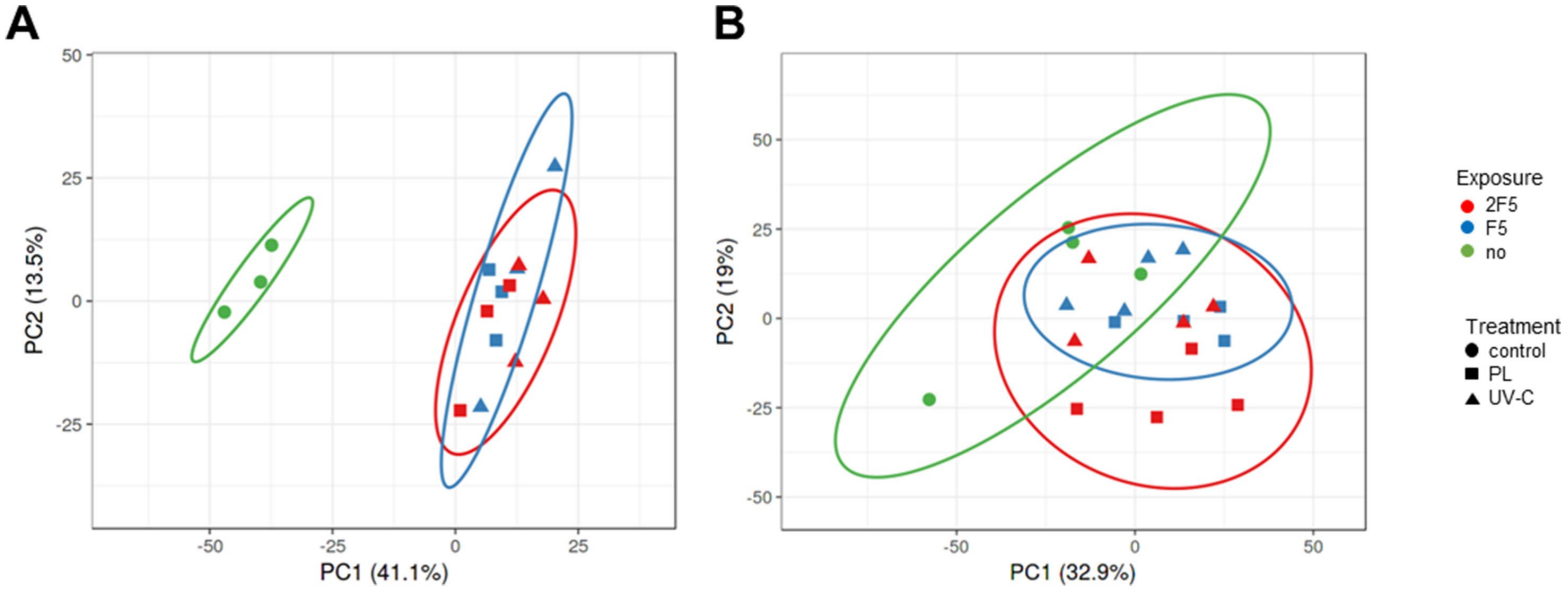
Principal component analysis (PCA) of *B. pumilus* proteomics data. **(A)** The PCA plots represent the 1,217 proteins identified in the three biological replicates for spores suspended in water. **(B)** The PCA plots represent the 1,357 proteins identified in the four biological replicates for spores sprayed on polystyrene (PS) Petri dishes. The axis labels indicate the percentage of total variance, explained by the first (PC1) and second components (PC2). Biological replicates for untreated spores (circle) are indicated in green, and biological replicates for spores treated with PL (square) and UV-C (triangle) are indicated in blue (*F*_5_) and red (2*F*_5_), respectively. *F*_5_ is the PL or UVC fluence allowing for approximately 5-log reduction. 2 *F*_5_ is 2 × *F*_5_, i.e., twice the fluence allowing a 5-log reduction.

The total proteome of the spores was then analyzed using tandem mass spectrometry (MS/MS) to assess the effects of irradiation on proteins. A total of 1,217 proteins were identified and their abundance established from 15 samples (3 biological replicates × 2 treatments (PL and UV-C) × 2 fluences (*F*_5_ and 2 *F*_5_), plus three untreated control samples) prepared from spores suspended in water (Supplementary Table S3). Initially, four biological replicates were obtained; however, due to technical issues, proteomic analysis was conducted on three replicates. Additionally, 1,357 proteins were identified and their abundance established from 20 samples (4 biological replicates) sprayed onto a PS Petri dishes (Supplementary Table S4). The number of proteins detected is consistent with our previous findings, in which 1,264 proteins were identified (Dorbani et al., 2024a). To obtain an overview of the proteomic variability between the treatments and exposures, a Principal Component Analysis (PCA) was performed (Figure 1). The PCA score plot of the first two principal components explained 54.6% of the total variance for spores suspended in water (Figure 1A). The first principal component (PC1) captured the largest variance of the data (41.1%), and evidences clear differentiation between untreated and PL- and UV-C samples. No separation of the samples based on the treatment and exposures, was observed even considering the second component (13.5% of the variability). Figure 1B shows that the PCA score plot of PC1 and PC2 accounted for 51.9% of the total variance for spores sprayed in PS Petri dishes, and did not separate PL and UV-C treatments from the untreated samples, whatever the exposure. Thus, our preliminary proteome comparisons did not reveal any substantial differences between the effects of UV-C and PL exposure on spores, whether they were suspended in water or sprayed on PS Petri dishes. However, PL and UV-C treatments significantly remodeled the proteome of spores when they were suspended in water, but not when they were sprayed on PS Petri dishes (Figure 1).

To identify proteins significantly influenced by treatment and exposure, we conducted pairwise comparisons of protein abundance between untreated spores and those treated with PL and UV-C. Specifically, proteins showing both a 1.5-fold change in abundance (|log_2_ fold-change| ≥ 0.59) and an adjusted *p*-value ≤ 0.05 were deemed affected by the PL or UV-C treatments (Supplementary Tables S3, S4). We then exclusively examined proteins exhibiting decreased abundance, as this decrease is indicative of their candidacy as targets of PL and/or UV-C.

#### Spores suspended in water

**Figure 2:**
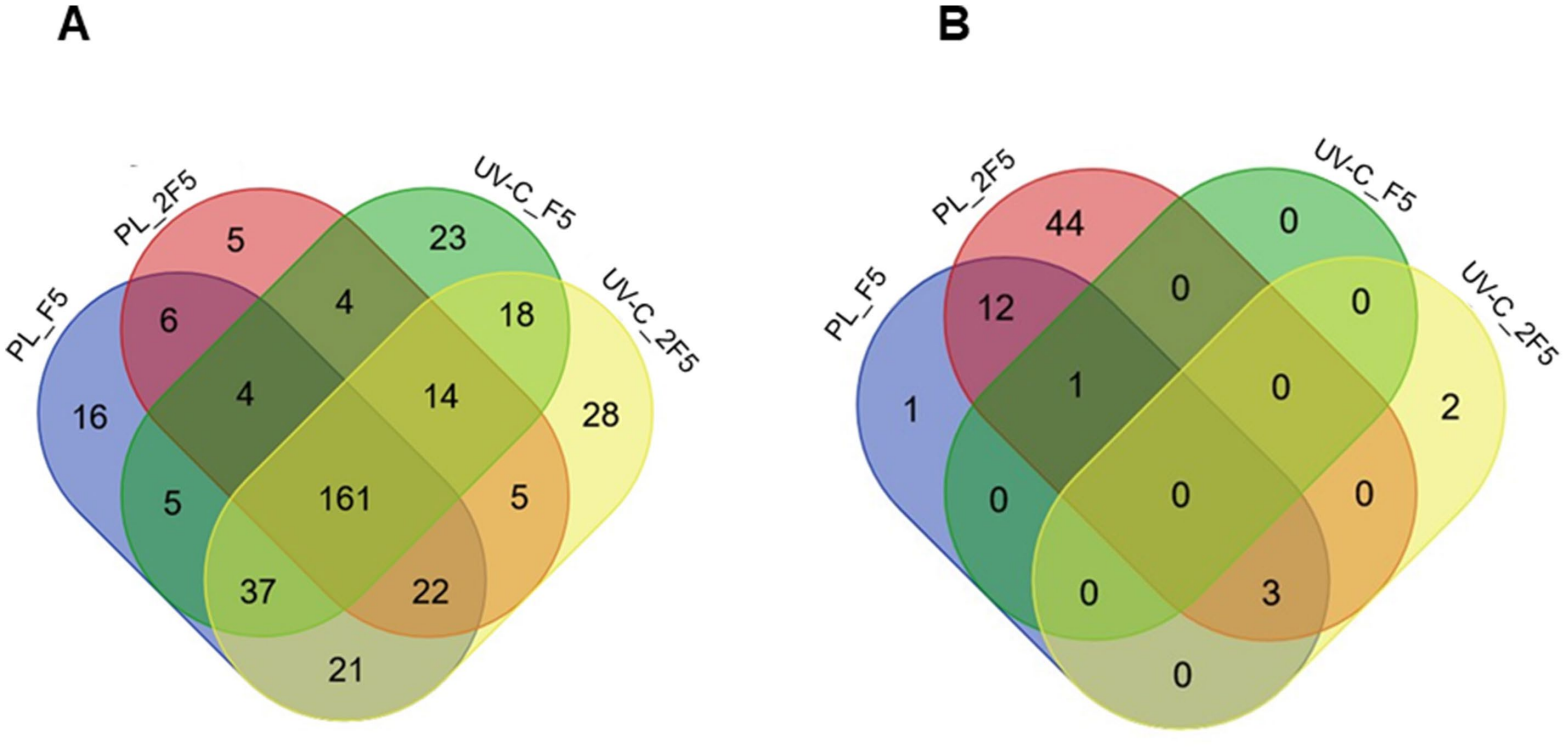
Venn diagrams of overlapping proteins with decreased abundance upon PL and UV-C treatments at *F*_5_ and 2*F*_5_ fluences. **(A)** The Venn diagram represents the distribution of the 369 proteins significantly depleted when spores are suspended in water. **(B)** The Venn diagram represents the 63 proteins significantly depleted when spores are sprayed on PS surface. *F*_5_ is the PL or UV-C fluence allowing for approximately 5-log reduction. 2*F*_5_ is 2 × *F*_5_, i.e., twice the fluence allowing a 5-log reduction.

Out of the 1,217 spore proteins identified by MS/MS, 369 showed a significant decrease in abundance levels (i.e., 30% of the spore proteome, Supplementary Table S3). The distribution of these 369 proteins according to treatment (UV-C and PL) and exposure (*F*_5_ and 2*F*_5_) was evaluated using a Venn diagram (Figure 2A). The data indicate that 300 proteins were targeted by PL, regardless of the exposure level. Among them, 273 were also targeted by UV-C, with 161 proteins being targeted by both treatments across the *F*_5_ and 2*F*_5_ exposures. Notably, none of these 161 proteins exhibited a significant change in abundance between PL and UV-C treatments, nor showed a significant decrease in abundance after exposure to 2*F*_5_ compared to *F*_5_. However, it is worth mentioning that 27 out of these 161 proteins became undetectable following *F*_5_ exposure (Supplementary Table S3).

#### Spores sprayed on PS Petri dishes

Out of the 1,357 identified spore proteins, only 63 (4.6%) showed decreased abundance. Among these 63 proteins, 57 were specifically targeted by PL, 4 were targeted by both PL and UV-C, and 2 were specifically targeted by UV-C. In the case of PL treatment, doubling the fluence is accompanied by a 4-fold increase in the number of proteins with decreased abundance (Figure 2B). Interestingly, the comparison of results obtained for spores on the PS Petri dishes and in suspension in water showed no common proteins regardless of the treatment. Taken together the results indicate that PL treatment has specific effects on a subset of spore proteins, rather than causing a widespread alteration of the entire proteome. This specificity is emphasized by the treatment intensity. Furthermore, the treatment environment plays a crucial role in the impact of PL on the spore proteome.

### Identification and characterization of PL-targeted proteins in *Bacillus pumilus* spores suspended in water

We focused on the 300 proteins, whose abundance decreased upon PL exposure (Supplementary Table S5). Among them, 46 are membrane-associated proteins, potentially localized in the inner membrane of the spores (Zheng et al., 2016). The other 254 proteins appear to be soluble proteins, mostly found in the spore core. However, we acknowledge that some proteins classified as soluble may also be associated with other spore structures, such as the coat or cortex. For example, YaaH, which has been identified in the spore coat of *Bacillus subtilis* (Imamura et al., 2010), is among these proteins. The membrane proteins primarily include transporters involved in various cellular processes and proteins engaged in cell wall biogenesis (Supplementary Table S5). Notably, two Duf-421 containing proteins (AIDKCPCB_02427 and AIDKCPCB_01829), potentially associated with spore resistance and germination (Yu et al., 2023), were significantly affected by both PL and UV-C treatments. The membrane protein most significantly depleted by PL was the YIEGIA-containing domain AIDKCPCB_02038. The gene encoding its homolog in *Bacillus subtilis* (YphB) is regulated by the sporulation transcription factor sigma-F. However, it is not essential for sporulation or germination (Decatur and Losick, 1996). To gain a deeper understanding of the functional annotation and characteristics of the 254 soluble proteins depleted by PL, we conducted an Ontology (GO)-based enrichment analysis.

**Figure 3:**
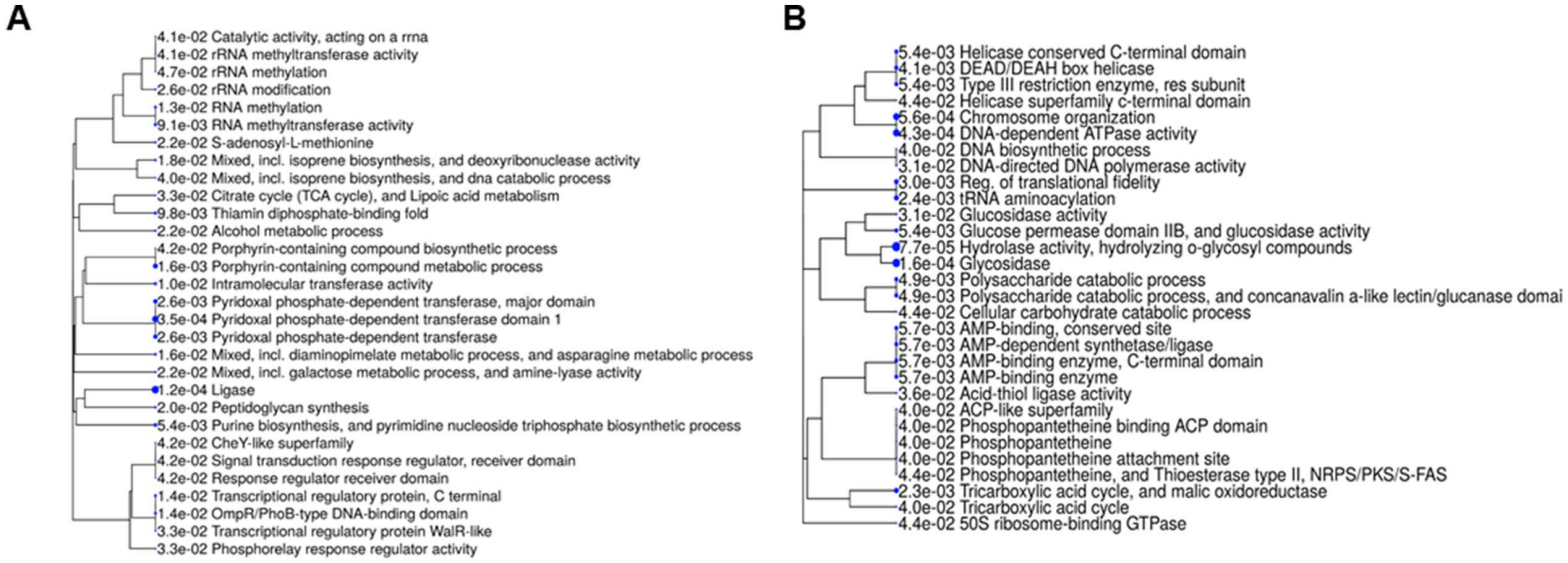
Gene Ontology enrichment analysis of spore core proteins depleted by PL treatment. **(A)** Spore core proteins exhibiting decreased abundance following PL treatment of spores suspended in water. **(B)** Spore core proteins exhibiting decreased abundance in PL-treated spores when spores are sprayed on PS Petri dishes. Significantly enriched biological pathways are highlighted using an interactive hierarchical clustering tree with ShinyGO v0.8.0. Biological pathways with many shared proteins are clustered together. Larger blue dots indicate more significant *p*-values. Pathways are listed in descending order of fold enrichment.

Figure 3A illustrates the top 30 GO terms and pathways, ranked by fold-enrichment score. The analysis revealed that the depleted proteins are involved in various metabolic processes, including catalytic, ligase, and transferase activities. Specifically, these processes encompass: (i) Various aspects of RNA metabolism, including rRNA and tRNA processing; (ii) Metabolic processes related to porphyrin-containing compounds (iii); Cell wall biogenesis; (iv) Central metabolism particularly the thiamine-dependent enzymes of the tricarboxylic acid cycle (TCA); (v) Signal transduction; (v) Nucleobase synthesis, DNA synthesis, and degradation, and repair. Among the proteins contributing to DNA synthesis, the chromosomal replication initiator protein DnaA stands out. In *B. subtilis*, DnaA is indispensable for governing and initiating genomic DNA replication (Kobayashi et al., 2003). Proteins associated with DNA repair include the DNA scanning integrity protein DisA, the RecA regulatory protein RecX, the DNA mismatch repair protein MutS, RadA, PolX, a spore photoproduct lyase (AIDKCPCB_01332), and the RuvA and RuvB proteins, which contribute to the survival of reviving spores following UV irradiations (Raguse et al., 2017). Notably, DisA, along with UvrA, and UvrB, became undetectable after *F*_5_ exposure (Supplementary Table S3).

**Figure 4:**
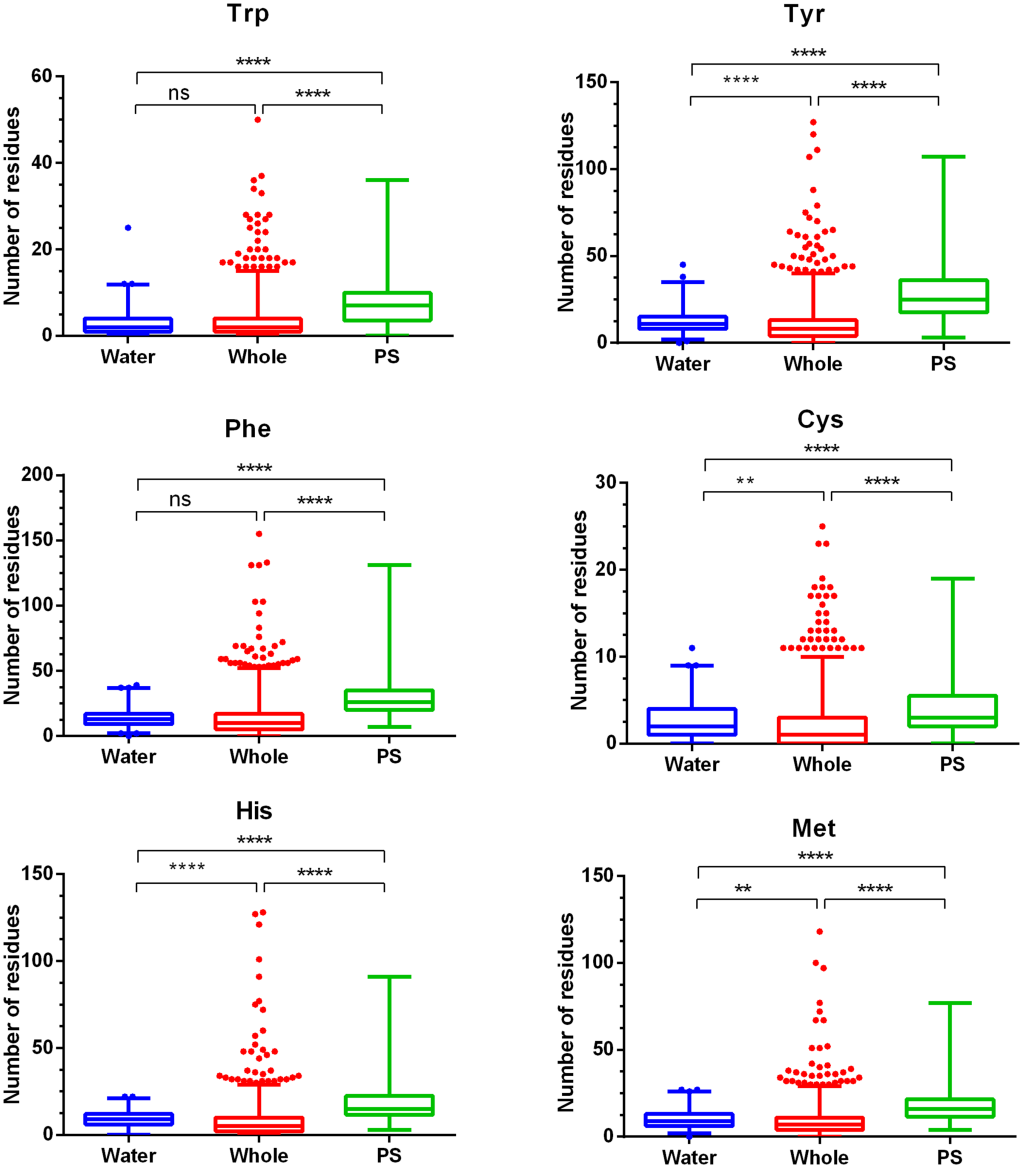
Comparison of photosensitive amino acid content in proteins depleted by PL treatment when suspended in water or sprayed onto PS Petri dishes, relative to the *B. pumilus* whole proteome. The analyzed amino acids include tryptophan (Trp), histidine (His), methionine (Met), phenylalanine (Phe), tyrosine (Tyr), and cysteine (Cys). Ns, no significant; **p-value < 0.01; ****p-value < 0.0001 based on one-way ANOVA and *post hoc* Tukey test.

Two proteins associated with protection/repair of RNA were also identified: the ribosome hibernation promoting factor, AIDKCPCB_03286, which, by associating with ribosomes, protects them from degradation (Helena-Bueno et al., 2024), and the tRNA nucleotidyltransferase CcA, which synthesizes and repairs the 3′-terminal CCA sequence of tRNA (Błaszczyk et al., 2023). It is worth noting that out of the 261 soluble proteins examined, only 4 were specific to spores, in addition to the spore photoproduct lyase (AIDKCPCB_01332): the germination protein YaaH (also known as SLeL, Christie and Setlow, 2020), characterized by its notably high decrease abundance level (Supplementary Table S3), a spore germination protein (AIDKCPCB_00024), a germination proteinase (AIDKCPCB_02306), and a putative protein involved in spore assembly envelope (AIDKCPCB_02308) (Abhyankar et al., 2019). Our data indicate that a notable proportion of the depleted proteins binds photosensitive compounds, such as porphyrins (Blanchon et al., 2023), thiamin (Aheika et al., 2021), and nucleobases (Barbatti et al., 2010), or are involved in their synthesis, suggesting their susceptibility to UV-induced photodamage. Protein photodamage may also result from the direct absorption of UV irradiation by specific amino acid residues, including tryptophan, tyrosine, phenylalanine, histidine, cysteine, and methionine, or through reactions with O_2_-induced intermediates (such as hydroperoxides) that involve these residues (Pattison et al., 2011). Therefore, we conducted an analysis of the composition of the 300 proteins depleted by PL in terms of these amino acids and compared the data with that from the whole proteome of *B. pumilus*. The analysis revealed that tyrosine, histidine, cysteine, and methionine residues exhibited a significant increase in abundance in the 300 PL-depleted proteins compared to the whole proteome, albeit with a moderate level of enrichment (Figure 4). This was not the case for tryptophan and phenylalanine residues.

The 27 proteins showing a significant decrease in abundance following PL exposure, but not following UV-C exposure, exhibit a diverse array of functions, although some are implicated in substrate transport, metabolic processes, and gene regulation (Supplementary Table S5). Moreover, their amino acid composition, and co-factor does not distinguish them significantly from the 261 proteins damaged by both PL and UV-C treatments (data not shown).

### Identification and characterization of PL-targeted proteins in *Bacillus pumilus* spores sprayed on PS Petri dishes

We focused on the 57 proteins depleted by PL exposure (Supplementary Table S6). Fourteen of them have been identified as membrane proteins. These are primarily transport proteins or proteins involved in peptidoglycan synthesis. One of them is the spore protein YabQ, (AIDKCPCB_00074), which is essential for the formation of spore cortex (Asai et al., 2001). The remaining 43 soluble proteins are mainly involved in DNA replication and genome stability and have associated activities such as helicase, exonuclease, and ligase. They also play roles in proteome control quality and central metabolism, with activities such as AMP and phosphopantetheine binding (Figure 3B). The proteins associated with DNA-related processes include the 3′ to 5′ helicase PriA, the error-prone translation synthesis DNA polymerase DnaE, and the proofreading 3′ → 5′ exonuclease DinG. These protein functions are crucial for overcoming replication stress (Carrasco et al., 2023) and potentially for repairing DNA damage (Matthews and Simmons, 2022). Additionally, the transcription-repair coupling factor Mfd was found to be depleted as a result of PL exposure (Table 1). Previous research has demonstrated its role in maintaining genomic stability by acting as an anti-mutagenic agent in spores exposed to harmful conditions, such as UV-C radiation (Ramírez-Guadiana et al., 2013). Interestingly, PriA and DnaE exhibited significant decrease abundance under both fluence conditions, while DinG and Mfd were only affected at the 2 *F*_5_ fluence level. Among the proteins specifically depleted following exposure to 2*F*_5_, we identified the protein RqcH, which plays a central role in the Ribosome Quality Control System (RQC). The RQC system manages errors occurring during gene expression or as a result of damaging agents, and it prevents the synthesis of proteins with various forms of aberrations (Filbeck et al., 2022).

**Table 1:** Changes in abundance of proteins involved in replication and UV-radiation repair mechanisms in PL-treated *Bacillus pumilus* spores at *F*_5_ and 2*F*_5_ fluences compared to untreated spores.

			Log_2_ (fold-change)	
Protein ID	Name	Description	*F*_5_	2 *F*_5_
Spores suspended in water				
AIDKCPCB_00001	DnaA	Chromosomal replication initiator protein	−2.48	−2.70
AIDKCPCB_00118	DisA	DNA integrity scanning protein	−2.42	−2.42
AIDKCPCB_02279	RecO	DNA repair protein O	−1.46	−1.46
AIDKCPCB_03286	Hpf	Ribosome hibernation promoting factor	−0.74	−1.74
AIDKCPCB_01997	Cca	CCA-adding enzyme	−1.68	−1.42
AIDKCPCB_01653	MutS	DNA mismatch repair protein	−1.42	−1.00
AIDKCPCB_00117	RadA	DNA repair protein	−1.35	−0.62
AIDKCPCB_00626	LigA	DNA ligase	−0.74	−0.60
AIDKCPCB_02434	RuvA	Holliday junction branch migration complex subunit A	−1.00	−1.00
AIDKCPCB_02433	RuvB	Holliday junction branch migration complex subunit B	−1.00	−1.00
AIDKCPCB_00785	RecX	Regulatory protein	NS^a^	−1.00
AIDKCPCB_01332	SplB	Spore photoproduct lyase	−0.77	−0.69
Spores sprayed on PS surface				
AIDKCPCB_02582	DnaE	DNA polymerase III subunit alpha	−0.79	−1.31
AIDKCPCB_01515	PriA	Primosomal protein N′	−0.84	−0.84
AIDKCPCB_00068	Mfd	Transcription-repair-coupling	NS	−0.78
AIDKCPCB_01992	DinG	3′-5′ exonuclease	NS	−0.74
AIDKCPCB_01508	RqcH	Rqc2 homolog RqcH	NS	−0.69

a NS: changes in abundance of proteins were considered not significant according to our criteria (absolute log 2 fold change ≥ 0.59, and *p*-value ≤ 0.05).

We then analyzed the composition of PL-damaged proteins, focusing on tryptophan, tyrosine, phenlyalanine, cysteine, and methionine, and compared them to the whole proteome of *B. pumilus*. The results showed a significant enrichment of all these amino acids in PL-targeted proteins compared to the entire proteome. Particularly noteworthy was the tryptophan enrichment factor exceeding 3, indicating a substantial increase in its presence in PL-targeted proteins (Figure 4).

## Discussion

Understanding the molecular impacts of PL holds significance in microbiology, given its efficacy in inactivating bacterial spores and its numerous applications for microbial safety or hygiene. The proteins within spores constitute pivotal targets in the inactivation procedures, where their degradation is frequently imperative to proficiently halt spore germination and/or outgrowth and ensure surface or matrix (medium) decontamination. In this study, we employed next-generation shotgun proteomics based on high-resolution tandem mass spectrometry to identify and quantify proteins, providing a detailed characterization of the protein landscape within the treated spores. Peptides detected in lower amount by MS/MS may be attributed to several factors, including a reduced abundance of the corresponding polypeptides in the analyzed fraction due to aggregation, precipitation, or proteolysis directly or indirectly induced by the treatment. Additionally, direct chemical modifications altering the global molecular weight of these peptides render their MS/MS spectra unsuitable for interpretation. Notably, our analysis did not reveal a decrease in the number of spectra assigned to peptide sequences in the treated spores compared to the control spores, thereby diminishing the significance of this latter explanation. Therefore, the methodology allows to monitor the molecular damages induced by irradiation at the proteome level. It is also important to note that the observed increase in abundance of some spore coat proteins (e.g., CotA, CotE, CotY, GerQ, and SpoIVA, Supplementary Table S3) does not reflect active upregulation but rather a relative enrichment. Since proteins were extracted immediately after treatment, there was no time for *de novo* protein synthesis. This apparent increase is therefore most likely due to the decreased abundance of treatment-targeted proteins, leading to a shift in the overall proteomic composition.

Our research demonstrates that when spores are suspended in water, PL irradiation significantly alters the proteome of *B. pumilus* spores, particularly depleting core proteins. In contrast to our previous study, poor degradation of spore coat proteins was observed; however, the strain and treatment conditions were different (Clair et al., 2020). Moreover, while more than 80 coat proteins have been identified in *B. subtilis* (Driks and Eichenberger, 2016), fewer than 10 were detected here. This may be due to differences in the *B. pumilus* spore coat composition, the limited annotation of *B. pumilus* proteins, or the intrinsic challenges of detecting highly insoluble proteins using proteomic approaches. In this study, the effect of PL on spores suspended in water closely resembles the effect observed with UV-C (254 nm) alone, implying that PL’s primary effect on the proteome stems from its UV-C component. However, when spores are deposited on a dry surface (PS Petri dish), the effect of PL on the proteome is considerably reduced, which can be attributed to the minimal impact of UV-C under these conditions, as UV-C alone shows almost no effect. Nevertheless, PL exhibits a unique effect on the proteome, as we observed more proteins being affected by PL than by UV-C alone, and the number of damaged proteins increases with higher inactivation rates under PL treatment.

The UV-C light penetrating a microorganism is directly absorbed by the DNA or RNA molecules inside vegetative cells or spores. This absorption results in the formation of cyclobutane dimers and single-strand breaks in the sugar–phosphate backbone of nucleic acids. These genetic changes hinder replication, transcription, and translation processes (Choudhary and Bandla, 2012). Nucleic acids in living cells are closely associated with a diverse array of proteins. Consequently, UV-C irradiation of cells can induce reactive interactions between DNA or RNA and the proteins in contact with them. One such reaction involves cross-linking between the amino acids in these associated proteins and the purine and pyrimidine free radicals (Stützer et al., 2020). Numerous proteins are known to form cross-links with nucleic acids, including those in close proximity to DNA due to their roles in genome structural organization, DNA polymerase, and proteins involved in DNA damage recognition and repair (Essawy and Campbell, 2024). We have demonstrated that these proteins are particularly depleted by both PL and UV-C treatment when spores are suspended in water, suggesting that nucleobase-protein cross-links may be responsible for the UV-C induced damage of several core proteins. Furthermore, our findings indicate that the depletion of core proteins by PL and UV-C may also occur due to their interaction with photosensitizers such as thiamine (Aheika et al., 2021), porphyrins and flavins, which have the potential to generate ROS in response to UV-C irradiation (Pattison et al., 2011). Direct absorption of UV-C photons by amino acids, particularly those containing aromatic rings like tryptophan, tyrosine, and phenylalanine, may also contribute to the decreased abundance of certain proteins. Furthermore, UV-C can generate free radicals that specifically oxidize cysteine and methionine residues in certain proteins, thereby contributing to their decreased abundance (Davies, 2005). When spores are spread on a PS Petri dish, they are dehydrated, which may affect their susceptibility to UV-C damage (Setlow, 2014b). In addition, the absence of water limits UV-C penetration, thereby reducing the extent of damage to biomolecules (Blau and Gallert, 2024). This difference could explain why PL treatment induces only minor changes in the proteome and primarily affects proteins that are not directly targeted by UV-C exposure. Interestingly, the core proteins specifically targeted by PL when spores are sprayed on PS Petri dishes are characterized by a higher content of photosensitive amino acids such as tryptophan, histidine, and tyrosine. This higher content may increase their susceptibility to damage by UV-B (Cai et al., 2013), which is known for its lower energy compared to UV-C. Additionally, these proteins have elevated levels of cysteine and methionine, which could enhance their susceptibility to damage through UV-A-induced oxidation (Karran and Brem, 2016).

How does proteome damage contribute to the rate of spore inactivation? No direct correlation was observed between the number of damaged proteins and the inactivation rate for spores suspended in water. However, it is possible that the presence of certain low abundance proteins crucial for nucleic acid repair and protection (Esbelin et al., 2016) may influence inactivation, though these proteins were not detectable at the 5-log inactivation level. On PS surfaces, the number of proteins impacted by PL was higher for a 7-log reduction compared to a 5-log reduction. In contrast, UV-C alone had a minimal impact on the proteome, despite inducing similar inactivation rates. This discrepancy suggests that while UV-C in PL contributes to spore inactivation, its effect on proteome is negligible, raising the question of how much protein damage contributes to the overall PL inactivation process.

## Conclusion

Our study provides new insights into the molecular impact of PL on bacterial spores, highlighting its distinct effect on the spore proteome depending on hydration conditions. We showed that PL significantly alters the proteome of *B. pumilus* spores in aqueous suspension, particularly by depleting core proteins involved in nucleic acid maintenance. This effect closely resembles that of UV-C 254 nm alone, suggesting that the UV-C component of PL is the primary driver of proteome modifications in liquid environment. On dry surface, however, the impact of PL on proteome is markedly reduced. Nevertheless, PL still induces proteome modifications beyond those observed with UV-C alone, particularly affecting proteins rich in photosensitive and oxidation-prone amino acids. These finding indicate that mechanisms beyond direct UV-C induced protein damage may contribute to PL-induced proteome alteration. Importantly, our results challenge the direct correlation between proteome damage and spore inactivation. While protein modifications were observed, their extent did not directly align with inactivation rates, suggesting that other factors - such as nucleic damage- may play a dominant role. Future investigations into specific post-translational modification, particularly oxidation events, will be critical to fully elucidate the contribution of protein damage to PL-induced spore inactivation.

## Data Availability

The datasets presented in this study can be found in online repositories. The names of the repository/repositories and accession number(s) can be found in the article/Supplementary material.

## Footnotes

^1^ http://www.uniprot.org/uniprot

^2^ https://string-db.org/

^3^ http://bioinfogp.cnb.csic.es/tools/venny/index.html

^4^ https://biit.cs.ut.ee/clustvis/

^5^ http://bioinformatics.sdstate.edu/go/
