# Supplementary material for: UV-C and hydration state drive pulsed light-induced proteome damage in Bacillus pumilus spores
Source: Front Microbiol. 2025 Apr 9;16:1579161. doi: 10.3389/fmicb.2025.1579161 (PMC12017682; doi:10.3389/fmicb.2025.1579161)
Supplement: Supplementary file 1 [file Table_1.DOCX]

**Table S1**. Inactivation (expressed as log reduction) of a water suspension of *Bacillus pumilus* spores treated by pulsed light (PL) at a 1.31 J/cm^2^ fluence (Fluence to 5-log reduction *F_5_*) and UV-C at 0.1 J/cm^2^ (*F_5_*) and at twice *F_5_* (*2F_5_*)

| Replication number | **Spore inactivation (log reduction) by the indicated treatment and at the indicated fluence** | | | |
| --- | --- | --- | --- | --- |
|  | PL | | UV-C | |
|  | *F_5_* | *2F_5_* | *F_5_* | *2F_5_* |
| #1 | 5.55 | >7.65 | 5.65 | >7.65 |
| #2 | 5.40 | >7.74 | 5.58 | >7.74 |
| #3 | 4.93 | >7.85 | 5.08 | >7.85 |
| #4 | 4.86 | >7.75 | 4.60 | >7.75 |
|  |  |  |  |  |
| Mean | 5.18 | >7.75 | 5.23 | >7.75 |
| Standard deviation | 0.34 | 0.08 | 0.49 | 0.08 |
