# Supplementary material for: UV-C and hydration state drive pulsed light-induced proteome damage in Bacillus pumilus spores
Source: Front Microbiol. 2025 Apr 9;16:1579161. doi: 10.3389/fmicb.2025.1579161 (PMC12017682; doi:10.3389/fmicb.2025.1579161)
Supplement: Supplementary file 2 [file Table_2.DOCX]

**Table S2**. Inactivation (expressed as log reduction) of a *Bacillus pumilus* spores sprayed on polystyrene and treated by pulsed light (PL) at a 1.24 J/cm^2^ fluence (Fluence to 5-log reduction *F_5_*) and UV-C at 0.08 J/cm^2^ (*F_5_*) and at twice *F5* (*2F_5_*)

| Replication number | **Spore inactivation (log reduction) by the indicated treatment and at the indicated fluence** | | | |
| --- | --- | --- | --- | --- |
|  | PL | | UV-C | |
|  | *F_5_* | *2F_5_* | *F_5_* | *2F_5_* |
| #1 | 5.01 | >7.42 | 4.96 | >6.85 |
| #2 | 5.45 | >7.45 | 5.22 | >7.00 |
| #3 | 4.83 | >7.51 | 5.29 | >7.32 |
| #4 | 5.49 | >7.42 | 5.26 | >6.88 |
|  |  |  |  |  |
| Mean | 5.20 | >7.45 | 5.18 | >7.01 |
| Standard deviation | 0.33 | 0.04 | 0.15 | 0.22 |
